# Generation of two successive attosecond pulses in separate spectral domains

**DOI:** 10.1038/s41598-020-64373-x

**Published:** 2020-04-30

**Authors:** Katalin Kovács, Valer Tosa

**Affiliations:** National Institute for R&D of Isotopic and Molecular Technologies, RO 400293 Cluj-Napoca, Romania

**Keywords:** Ultrafast lasers, High-harmonic generation

## Abstract

We demonstrate that two different single attosecond pulses (SAP) can be obtained from naturally separated spectral domains formed during high-order harmonic generation and propagation in a gas medium. We propose a feasible experimental configuration in which one can obtain an SAP in a lower energy domain (<300 eV), or another SAP in a higher energy domain (>300 eV). Without filtering, a double attosecond pulse emission with fixed temporal separation is obtained. The gap between the two spectral domains is close to the onset of the water window.

## Introduction

High-order harmonic generation (HHG) with near-infrared (NIR) and mid-infrared (MIR) driving laser pulses is the well-established table-top method to obtain highly coherent pulses in the extreme ultraviolet (XUV) and soft X-ray spectral domain with temporal width in the order of tens-to-hundreds of attoseconds^1,2^, paving the way for the development of Attosecond Science^3,4^. Applications of the attosecond pulses range from studying the real-time electron dynamics in atoms and molecules, to strong-field induced processes in solids or biomolecular imaging^5–9^. For the applications it is usually required that the attosecond pulses should be bright, reach high energy like the water window (282–533 eV), and in many cases it should be only one isolated attosecond pulse^10^. One important research challenge is to elaborate methods to control the generation and characteristics of attosecond pulses^11^.

The maximum photon energy obtainable in HHG is given by the cutoff-law ${E}_{cut}={I}_{p}+3.17\,\cdot \,{U}_{p}$ where ${U}_{p} \sim {E}^{2}\,\cdot \,{\lambda }^{2}$ is the ponderomotive energy of the electron. Due to the ${\lambda }^{2}$ scaling MIR driving pulses are advantageous because the available photon energy can be extended to the water window. However, due to the wave packet spreading in the continuum the efficiency of HHG scales as ${\lambda }^{-\mathrm{(5}-\mathrm{6)}}$^12^. HHG by long wavelength pulses has gained attention since the laser technology has evolved and ultrashort, carrier-to-envelop phase (CEP) stable laser pulses at high intensity are available also in the MIR spectral domain^2,13–21^.

As known, with few-cycle driving pulses, there exists the possibility to obtain experimentally one single attosecond pulse (SAP)^10^. However, whenever in an experiment a quasi-continuous high-harmonic spectrum is measured, a basic question arises: is this the signature of a SAP, or the quasi-continuous spectrum is the superposition of different radial components emitted shifted in time? When the driving pulse is very short (few-cycle) and very intense, ionization gating^22^ may occur, and an SAP is obtained. In the same time the high-harmonic spectrum strongly depends on the CEP of the driving pulse^23,24^: with changing CEP the harmonic spectrum changes from continuous to modulated and indicates the change from one single emission to interference between two successive emissions. Quantum path analysis reveals that the short and long trajectory components are influenced differently by the change in the driving field’s CEP^25,26^.

In this work we demonstrate that the total macroscopic spectrum can be generated in two distinct spectral regions: a low energy one corresponding to short trajectory emission in the plateau region and a high energy one corresponding to cutoff trajectories. We show that these two regions correspond to two separated bursts in time, so that by spectral filtering one can select the burst of low energy, the other burst of high energy, or keep both bursts with fixed delay. We show that by varying the CEP we can vary the relative intensity of the two bursts, and we identify the experimental parameters that act like “control knobs”. We also demonstrate the stability of the proposed configuration against the driving pulse energy and gas pressure. The configuration we investigate is not optimized for SAP generation, but rather we concentrate on the temporal and spectral separation of the double pulse structure.

## Modeling Tools and Proposed Experimental Configuration

### Model

The numerical simulations have been carried out using the extended version of the 3D non-adiabatic model presented in^27^. In this framework we first solve the pulse propagation in ionized gas medium and then calculate the atomic dipole induced by the driving field using the strong-field approximation (SFA)^28^. The macroscopic harmonic signal is the result of the coherent superposition of the single dipole emissions in each spatial point. The interaction region has cylindrical symmetry in the simulations. Since the model has been described in several papers^27,29–31^, we summarize here the main steps of the calculation.

1. The propagation of the fundamental laser pulse in a gaseous medium is very important to be accurately treated^32^. The non-linear wave equation for the carrier ${E}_{l}(r,z,t)$ of the driving field is solved:
  1 $${\nabla }^{2}{E}_{l}(r,z,t)-\frac{1}{{c}^{2}}\frac{{\partial }^{2}{E}_{l}(r,z,t)}{\partial {t}^{2}}=\frac{{\omega }^{2}}{{c}^{2}}(1-{\eta }_{{\rm{eff}}}^{2}(r,z,t)){E}_{l}(r,z,t)\,,$$
  The expression for the space-time dependent refractive index of the medium is:
  2 $${\eta }_{{\rm{eff}}}(r,z,t)={\eta }_{1}(r,z,t)+{\eta }_{2}I(r,z,t)-\frac{{\omega }_{p}^{2}(r,z,t)}{2{\omega }^{2}},$$
  where the first linear term ${\eta }_{1}=1+{\delta }_{1}-i{\beta }_{1}$ contains the dispersion and absorption of neutrals; the second term accounts for the optical Kerr effect dependent on the nonlinear refractive index (${\eta }_{2}$) and laser intensity ($I(r,z,t)$); the last term expresses the plasma dispersion, with plasma frequency ${\omega }_{p}=(4\pi {e}^{2}{n}_{e}/m{)}^{\mathrm{1/2}}$, ${n}_{e}$ is the free electron number density, $e$, $m$ are the electron charge and mass, $\omega$ is the driving pulse’s central frequency. For the calculation of the ionization rate of He we use the model from^33^ and take into account the depletion of the ground state. The non-trivial spatial and temporal variation of the refractive index induces the reshaping of the driving pulse. The ionization front of a strong laser pulse induces rapid variation of the medium’s refractive index, and thus the pulse itself suffers distortions in its spatial shape as well as in its temporal and spectral properties^29,30^.
2. The elementary interaction between the driving pulse and individual atoms is treated within the SFA framework. The nonlinear dipole which is the source of the harmonic radiation is calculated by solving the Lewenstein integral^28^. The single dipole will be calculated in each spatial point using the *propagated* pulse, therefore the harmonic dipole radiation inherently contains the phase properties of the modified laser field. The radial variation of the driving pulse implies that all intensity-dependent terms will be non-homogeneous radially. This radial non-homogeneity of the propagated fundamental pulse will be reflected in the whole process of HHG.
3. The nonlinear dipole radiations are sources for the macroscopic harmonic field which builds up propagating in the same gas medium. Absorption and dispersion of the harmonic field are taken into account. The propagation equation for the harmonic field has similar structure as Eq. 1, with the source term ${P}_{{\rm{nl}}}(t)$ which is the non-linear polarization calculated in step 2:
  3 $${\nabla }^{2}{E}_{h}(r,z,t)-\frac{1}{{c}^{2}}\frac{{\partial }^{2}{E}_{h}(r,z,t)}{\partial {t}^{2}}={\mu }_{0}\frac{{{\rm{d}}}^{2}{P}_{{\rm{nl}}}(t)}{{\rm{d}}{t}^{2}},$$
4. The simulation method has been extended to include specific experimental conditions, and to better elucidate the underlying physics. (i) The model gives the possibility to work with different beam types, for example Gaussian, truncated Gaussian, super-Gaussian or Bessel. (ii) The interaction medium where HHG takes place can be moved along the optical axis, and a distribution of the gas density can be included. (iii) The generated harmonic field can be refocused and/or clipped with an iris. The Hankel transform is used to calculate the signal in the far-field (i.e. detection plane), knowing the harmonic near-field. The radial and temporal profile of the total harmonic field or selected harmonics can be calculated. (iv) Quantum trajectory analysis^34^ was used as an independent verification of the temporal and phase properties of attosecond pulses at sub-optical-cycle temporal resolution. The method is based on numerically solving the complex-valued saddle-point equations^35,36^ in the case of arbitrary pulse shape, for example the propagated and distorted driving pulse for the present study. The validity of the saddle-point approach in this context is supported also in the recent review^37^.

### Proposed experimental configuration

The main goal of this paper is to demonstrate that in an experimentally feasible configuration one can obtain two successive attosecond pulses which are generated by two naturally separated spectral regions. For this purpose we constructed a case with experimental layout and parameters similar to those reported in Fig. 1 of ref. ^15^. It is important to note that this is a typical configuration for HHG, we do not impose any special experimental conditions. The driving wavelength of $\lambda =1700$ nm has the advantage that it allows for extended single-dipole cutoff; the extremely short duration of 6 fs ensures that we will have only few emission events per pulse; the 9 mm long gas medium contains He, at 200 Torr pressure and is placed in the converging beam, such that it ends in the nominal laser focus position (*f* = 50 cm). A pulse of *E*_pulse_ = 1 mJ, creates *I*_peak_ = 9 × 10^14^ W/cm^2^ peak intensity at the beginning of the gas cell.

**Figure 1:**
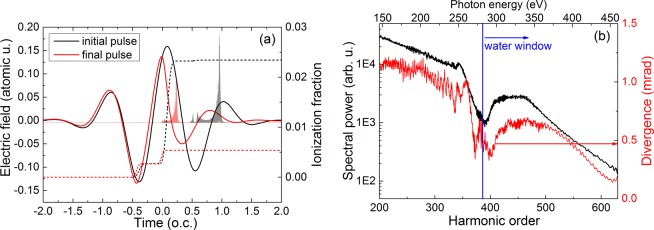
(**a**) Driving pulse shape (solid line) along with the temporal ionization dynamics (dashed line) at the beginning (black) and at the end (red) of the propagation in 200 Torr He. Shaded in the background we show the dipole radiation filtered between harmonic orders H200–630. Time is expressed throughout the paper in units of the driving field’s optical cycle ($T$), the nominal pulse peak is time zero. (**b**) (black curve, left axis) Radially integrated harmonic power spectrum at the end of the interaction region. The blue vertical line indicates the beginning of the water window spectral domain at 282 eV. (red curve, right axis) Radially averaged divergence of harmonics at the medium exit.

In order to verify the existence and validity range of the spectral separation, we varied some of the above parameters: CEP of the driving pulse, the input pulse energy i.e. the peak intensity in the input plane, as well as the gas pressure in the interaction region. For most of the applications the refocusing of the XUV is desired; for this purpose we also address the focusability of the attosecond pulses.

## Results and discussion

### Driving pulse propagation and harmonic spectrum

In Fig. 1(a) we show the temporal shape of the driving laser pulse at the beginning and at the end of propagation with black and red lines, respectively. The ionization dynamics during the pulse is also shown, with black dashed (initial) and red dashed (final) lines. The well known self-phase-modulation and the decrease of the peak intensity are clearly present due to the steep ionization front around the peak of the pulse. This effect scales with ${\lambda }^{2}$, therefore longer wavelength pulses are particularly affected even by a relatively low ionization level, which in this particular case is between 2.3% and 0.5%. The attosecond pulse emissions shown shaded in the background indicate that two main events take place during the laser pulse: (1) the earlier ($T\approx 0.2$) burst is due to the recombination of electrons ionized around $T=-\,0.3$; this emission can contain all spectral components up to the cutoff. (2) The second burst at $T\approx 0.6$ is due to recombining electrons which were ionized right after the pulse peak.

Figure 1(b) shows the radially integrated harmonic power spectrum at the exit of the interaction region, between harmonic orders 200 and 630 (**H200–H630**) which corresponds to **146–460 eV**. After 250 eV we observe a gap in the spectral power, or in other words we have a double plateau structure: a first cutoff around 255 eV, then a revival at higher photon energies 300–365 eV. The gap in the spectrum is at the beginning of the water window region which could be essential for applications in soft x-ray microscopy. The average divergence of the harmonics at the medium exit is also represented in Fig. 1(b) as a function of photon energy. It follows closely the power spectrum dependence, being around 1 mrad in the low energy region and around 0.5 mrad in the high energy domain. These values prove a very good collimation of the harmonics and will be later correlated with the spectral-spatial structure of the focused far-field.

Since the spectrum in Fig. 1(b) is a volume integrated quantity it is essential to examine the radial structure of the harmonic field at the medium exit. In the next subsection we will explore the radial structure of the harmonic fields, the main goal being to demonstrate the spectral separation of the two attosecond pulses, as well as to identify the “control knobs” which switch on/off this spectral separation.

### Spectrally separated attosecond pulses

**Figure 2:**
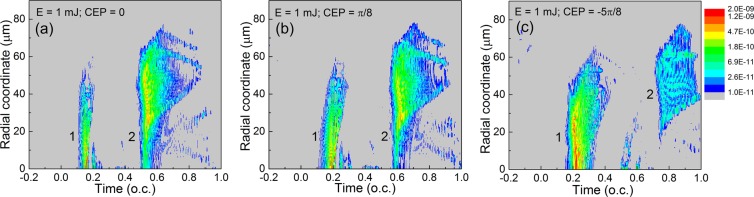
Temporal–radial maps of the attosecond pulses in the H200–H630 spectral domain. Snapshots are taken at the exit of the interaction region. Color scale spans two orders of magnitude, logarithmic scale, arbitrary units. In panel (**a**) CEP = 0; (**b**) CEP = *π*/8; (**c**) CEP = −5*π*/8.

In Fig. 2 we show the temporal–radial maps of the attosecond pulses filtered in the H200–H630 spectral domain. The snapshots are taken at the exit of the interaction region, for different CEP values of the driving pulse. With reference to the single-dipole emissions shown in Fig. 1(a) we observe that the double pulse structure has been maintained during propagation, but the long trajectory components of the second emission are not present in the final macroscopic harmonic field as they did not find good phase matching conditions during propagation. For future reference we labelled the earlier and later emissions with 1 and 2, respectively.

**Figure 3:**
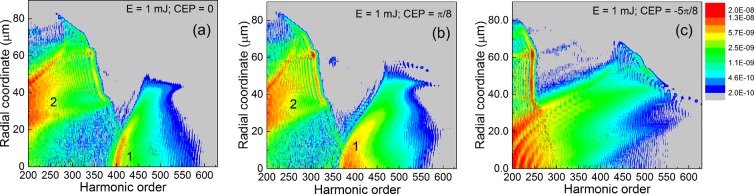
Radial maps of the harmonic radiation at the exit of the interaction region. The three cases are identical to those from Fig. 2. Snapshots are taken at the exit of the interaction region. Color scale spans two orders of magnitude, logarithmic scale, arbitrary units.

In Fig. 3 we show the radial map of harmonics in the H200–H630 spectral region, also at the exit of the medium, in the same three cases of different CEP values of the driving pulse as presented in Fig. 2. The temporal–radial and the spectral–radial maps are complementary to each other, they are two projections of a three-dimensional parameter space with temporal, spectral and radial coordinates. Therefore, the information contained in Figs. 2 and 3 have to be interpreted in pairs.

In Fig. 2(a) we observe that emission 1 is developed close to the axis, while emission 2 has the main contribution off-axis, in a ring of $r\in [20,50]$ $\mu m$. Comparing this feature with Fig. 3(a), we can clearly identify that emission 1 is associated with higher orders having the maximum at H400–H420. Emission 2 contains lower orders up to H350, maximum being around H200–H250. The spectral separation of the attosecond pulses 1 and 2 is visible in Fig. 3(a), the gap is around H380 (277 eV). The maps are qualitatively similar for a slightly modified CEP, as shown in Figs. 2(b) and 3(b) where CEP = *π*/8. The spectral separation of the two attosecond pulses still persists, but the gap between the frequency domains moved to lower orders, namely around H350 (255 eV). When we modified the driving pulse’s CEP to −5*π*/8, as seen in Fig. 3(c), the spectral separation does not happen, emissions 1 and 2 labeled in Fig. 2(c) cannot be identified in the corresponding frequency map in Fig. 3(c).

The results so far indicate that (1) the spectral separation is CEP-dependent; (2) the spectral gap can be slightly controlled by the CEP of the driving pulse.

**Figure 4:**
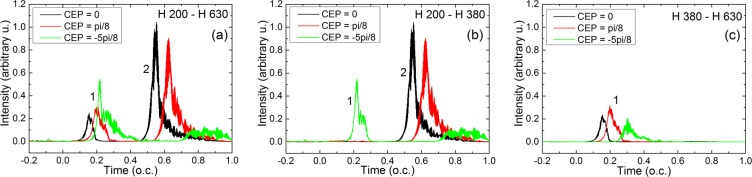
Radially integrated attosecond pulses in the far-field. (**a**) Full spectrum between H200–H630. (**b**) The lower part of the spectrum between H200–H380. (**c**) The high part of the spectrum between H380–H630. In each panel the colors identify the three cases: (black) CEP = 0; (red) CEP = *π*/8; (green) CEP = −5*π*/8.

The combined information contained in the panels of Figs. 2 and 3 is helpful, but we need evidence of the temporal AND spectral separation of the attosecond pulses, simultaneously. In simpler terms: we need to verify that the emission 1 contains only high-order spectral components, while emission 2 is composed only by lower order harmonics. The final confirmation is given in Fig. 4, where we show for the three cases the radially integrated attosecond pulses in the far-field, placed 1 m in vacuum from the medium, assuming no focusing.

When the full spectrum is inverse Fourier transformed, the double attosecond pulse structure is visible for all CEP values, see Fig. 4(a). The properties of the double pulse for the cases with CEP = 0 and CEP = *π*/8 are qualitatively similar, there is a fixed delay of $\tau =0.4$ T (2.26 fs) between emissions 1 and 2. The intensity ratio ${I}_{1}/{I}_{2}$ of emission 1 to emission 2 are: ${I}_{1}/{I}_{2}=0.24$ for CEP = 0, and ${I}_{1}/{I}_{2}=0.33$ for CEP = *π*/8.

When a low-pass spectral filter is applied such to keep only radiation below H380 (277 eV), as seen in Fig. 4(b) emission 2 is conserved with full intensity in cases with CEP = 0 and CEP = *π*/8, and a strong single attosecond pulse is obtained. The CEP = −5*π*/8 keeps the temporal separation, however, both emissions 1 and 2 contain the low order components. This result is in perfect agreement with the spectral – radial map in Fig. 3(c).

Further, if our filter is high-pass and we keep only the high-energy part of the spectrum with harmonics above H380 (>277 eV) a single attosecond pulse from emission 1 is generated in all three cases, as shown in Fig. 4(c).

It is of course important to address the focusability of the obtained attosecond pulses, as this is required in most pump-probe experiments. As shown in^38,39^, when focusing shorter and shorter pulses, the spatial and spectral/temporal configurations in the focal region increasingly deviate from the known configurations of continuous waves, even in the absence of dispersive focusing elements. Here we assume that the harmonic field is focused by an ideal non-dispersive element with f = 0.5 m placed at 1 m distance from medium. We then analysed the temporal–radial structure of the far-field in a $2f-2f$ configuration.

**Figure 5:**
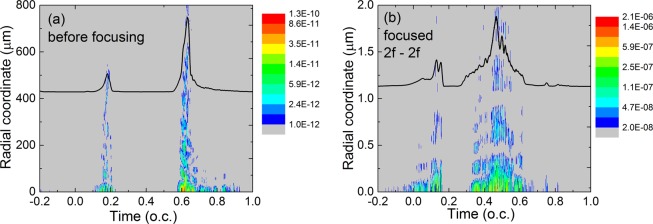
(**a**) Temporal–radial profile of the attosecond bursts in the far-field at 1 m distance from the exit of the interaction region, as detected without focusing. The black curve on top of the map is the radially integrated and smoothed attosecond pulse. (**b**) At 1 m distance a focusing mirror with f = 50 cm was placed and the attosecond bursts detected in $2f-2f$ configuration. The black curve on top of the map is the radially integrated and smoothed attosecond pulse.

In Fig. 5(a) we show the temporal – radial structure of the HHG field just before the focusing element, and in Fig. 5(b) the same quantity at the focus. First we have to mention that in all cases we investigated, the spectral separation observed in the near-field is preserved in the far-field. Moreover, the ring structure observed in the near-field at low orders is present also in the far-field, but the intensity is maximum on-axis, similar to the structures reported experimentally in^40,41^. Second, due to the low divergence of the harmonics (see Fig. 1b) we obtain a good collimation around beam axis, even for the burst 2 which was generated off-axis in the near-field. Spectral distribution within the two domains will be changed to some extent due to the known effects of frequency dependent diffraction as blue spectral components are focused more tightly than red ones^38^. Comparing the far-field temporal profile of the bursts before and after focusing we note that the main effect is their temporal dispersion and shift. For example, the focused burst 1 extends between 0 and 0.2 optical cycles (o.c.) while burst 2 extends from 0.3 to 0.8 o.c. when focused, compared to the much shorter burst before focusing (see the inset pulse shapes in Fig. 5(a,b)). The same effect was reported in^38^ when focusing a sub-cycle pulse (1.8 fs at 800 nm) by a mirror of 50 cm focal length. This effect might be detrimental for harmonic peak intensity, but we must stress that the two bursts are still generated by the two separated spectral regions so that in a pump-probe experiment the temporal dispersion is not necessarily a disadvantage. Further, we compared the brightness of the emission 2 (which contains the plateau harmonics) with and without focusing. Despite the fact that focusing induces temporal lengthening of this emission, the focused spot size is small enough to finally result in a 100 times enhanced brightness. Based on this calculation we conclude that XUV refocusing is possible even for the plateau harmonics and recommended for further applications.

### Mechanism of spectral separation

Next, we want to explain the spectral separation of the two emissions. First, it is obvious that the spectral components in emission 2 cannot contain very high-order harmonics due to the reduced average intensity of the driving pulse in the trailing edge. The question remains: what causes that in emission 1 only the high energy components are present after propagation? Indeed, it is not evident why for certain CEP values the lower orders are absent from emission 1.

**Figure 6:**
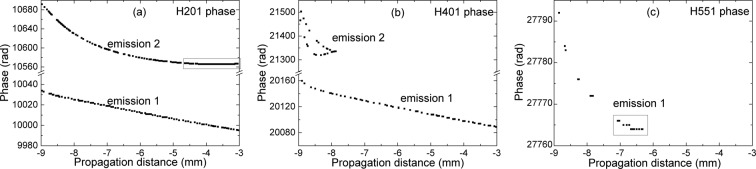
(**a**) Phase of H201 as it varies along propagation axis in emissions 1 and 2. The rectangle emphasizes the section where the harmonic phase variation is $< \pi$. (**b**) Phase of H401 as it varies along propagation in emissions 1 and 2. The lack of constant phase zone hinders the coherent construction of H401. (**c**) Phase of H551 as it varies along propagation in emission 1. The rectangle emphasizes the section along propagation where the harmonic phase has $< \pi$ rad variation.

Trajectory analysis gives the answer for this question. We studied in detail the case ${I}_{peak}=9\,\cdot \,{10}^{14}$ W/cm^2^, CEP = 0, and calculated the phases of trajectories responsible for harmonics H201 (present only in emission 2), H401 (in the spectral gap) and H551 (present only in emission 1), as presented in Fig. 6. The results emphasize the crucial role of the propagation effects which determine the final macroscopic harmonic signal. H201 is clearly present at dipole level in both emissions 1 and 2, which is confirmed by the quantum trajectory calculations and shown in Fig. 6(a). However, in emission 1 the phase of this harmonic varies significantly along propagation, $\approx$ 40 rad during the 9 mm medium. In emission 2 the trajectory responsible for H201 benefited from almost constant phase in the $z\in [-\mathrm{5;}-\mathrm{3]}$ mm section, and therefore could build up constructively. Harmonics in the spectral gap, in particular H401 examined by us (Fig. 6(b)), have strong phase variation all along propagation, making it impossible to grow in intensity. On the other hand, harmonic H551 (Fig. 6(c)) in emission 1 had $< \pi$ phase variation in the $z\in [-\mathrm{7;}-\mathrm{6]}$ mm portion and in this section the radiation could build up constructively. Trajectory analysis confirms again, that important aspects of the HHG process are basically influenced by propagation effects, therefore macroscopic modeling of HHG is essential.

### Validity range of spectral separation

Up to this point we identified one “control knob” for the spectral separation of the high-harmonic emissions, which is the CEP of the driving pulse. Due to the fact that the mechanism of HHG and consequently the possible spectral separation occurs at sub-optical-cycle time scale, this process is CEP-sensitive. In addition, for the feasibility of the experimental realization it is important to explore the (macroscopic) parameter space in which the spectral separation of the two successive attosecond pulses is still conserved. For this purpose we varied the input laser pulse **energy** and the **pressure** in the generation medium. We consider that fluctuation of the input pulse energy is a common issue, while the gas pressure is an easily tunable parameter in HHG experiments.

**Figure 7:**
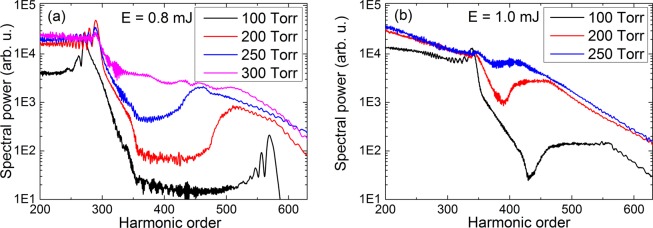
(**a**) Harmonic spectra obtained with E = 0.8 mJ laser pulse energy and pressure values p = 100; 200; 250; 300 Torr, respectively. (**b**) Harmonic spectra obtained with E = 1 mJ laser pulse energy and pressure values p = 100; 200; 250 Torr, respectively.

In Fig. 7 we summarize the results obtained for various pulse energy and pressure values. Generally we can observe that the spectral gap is present for most of the pulse energy – gas pressure combinations. More precisely, at 0.8 mJ pulse energy (this yields $7\cdot {10}^{14}$ W/cm^2^ peak intensity at the cell entrance) the spectral separation is present up to 250 Torr pressure of He, while at 1 mJ pulse energy the separation is visible up to 200 Torr. We performed these simulations with CEP = 0, and the corresponding temporal – radial and spectral – radial maps are qualitatively similar to those presented in Figs. 2 and 3, and confirms the spectral separation of the two successive attosecond pulses. At fixed input pulse energy the spectral gap moves toward lower orders as the pressure is increased, until the gap becomes a second plateau. This occurs at 300 Torr for the 0.8 mJ pulse energy, while at 250 Torr for the 1 mJ case. The reason for the disappearance of the spectral gap is that as pressure increases in the medium this induces higher plasma density due to ionization. Higher plasma density causes stronger defocusing of the driving field, resulting in decreased high-harmonic cutoff. Cutoff is reduced to such extent that the cutoff harmonic orders in emission 1 spectrally overlap with the plateau harmonics from emission 2. We also verified the phenomenon for even higher pulse energy, namely 1.2 mJ, but the radial chirp of the harmonics is so strong that it blurs the spectral gap.

## Conclusions

In conclusion, we numerically demonstrate that high harmonics generated in He gas by few cycle MIR pulses are emitted in two separated spectral domains formed during propagation in a long gas medium. We also show that these two spectral domains are emitted in two separate pulses so that by filtering it is possible to select one of them or both.

From the fundamental point of view, we emphasized the importance of the macroscopic propagation effects in shaping the final XUV emission. The phenomenon described here as spectral separation of successive attosecond pulses can be an example of space-time coupling in nonlinear optics, good candidate to be further explored and exploited experimentally.

From the practical point of view, we proposed a HHG configuration based on a feasible experimental setup with the main advantage that it allows the generation of two attosecond pulses having separate spectral content, but both in the XUV regime. In this configuration one can keep the total flux of one emission, which is an advantage knowing the low efficiency of the HHG process especially with increasing driving wavelength^12^.

Further investigations are ongoing to explore such generation schemes using NIR pulses and/or higher pulse energies.
